# Acromio-Humeral Distance Is Associated with Shoulder External Strength in National Elite Badminton Players—A Preliminary Study

**DOI:** 10.3390/sports9040048

**Published:** 2021-03-31

**Authors:** Simon Vadstrup Schmidt, Jannik Andersen Engelhardt, Ann Cools, Stig Peter Magnusson, Christian Couppé

**Affiliations:** 1 Institute of Sports Medicine Copenhagen, Department of Orthopaedic Surgery M, Copenhagen University Hospital—Bispebjerg and Frederiksberg, 2400 Copehagen, Denmark; vadstrups@gmail.com (S.V.S.); jengelhardt89@gmail.com (J.A.E.); ann.cools@ugent.be (A.C.); p.magnusson@sund.ku.dk (S.P.M.); 2 Center for Healthy Aging, Institute for Clinical Medicine, University of Copenhagen, 2200 Copenhagen, Denmark; 3 Department of Physical and Occupational Therapy, Copenhagen University Hospital—Bispebjerg and Frederiksberg, 2400 Copenhagen, Denmark; 4 Department of Rehabilitation Sciences, Ghent University, 9000 Ghent, Belgium

**Keywords:** overhead athlete, sport-specific adaptations, shoulder rotational strength, ultrasound, supraspinatus tendon hypertrophy

## Abstract

Purpose: To examine acromio-humeral distance (AHD) and shoulder isometric strength for external rotation (ER) and internal rotation (IR) in national elite badminton players. Methods: Seven elite badminton players with asymptomatic shoulders aged 24 ± 4 (mean ± SD) from the Danish national badminton team were investigated. Shoulder AHD, isometric strength in ER and IR were bilaterally assessed with ultrasonography and a hand-held dynamometer (HHD). Results: AHD was greater on the dominant vs. the nondominant side (*p* = 0.018). Moreover, IR strength was greater on the dominant side vs. the nondominant side (*p* = 0.041). Furthermore, AHD and ER strength were highly correlated on the dominant side (*p* = 0.007, *r* = 0.900). A correlation was also shown between AHD and the ER/IR strength ratio on the dominant side (*p* = 0.033, *r* = 0.793). Conclusion: This preliminary study demonstrates that shoulder ER strength is strongly associated with AHD size, largely reflecting supraspinatus tendon-muscle hypertrophy as a result of sport-specific adaptation in national elite badminton players with asymptomatic shoulders. These novel data also suggest that habitual loading of the shoulder improves the supraspinatus tendon size, which may lower the mechanical stress and potentially reduce the risk of injury. This warrants strengthening the shoulder external rotators as a potential strategy to reduce the risk of future shoulder injury.

## 1. Introduction

Shoulder overuse injuries are common in badminton players [1]. Studies have reported that 52% of both recreational and elite badminton players have an earlier or current shoulder injury [2,3]. Both incidence and prevalence are substantially higher than in the general population [4].

Electromyographical recordings and movement analysis have demonstrated that the act of throwing has excellent similarity in overhead sports, both with and without a racket [5]. During the act of throwing, the eccentric forces generated put excessive stress on the rotator cuff tendons and muscles, the capsule and the ligamentous structures of the shoulder [5]. It is well established that in overhead sports the strength of the internal rotator muscles is typically greater on the dominant side compared with the nondominant side without an accompanying increase in external rotational strength [6,7,8,9]. These adaptations, including shoulder external rotation weakness, have been linked to an increased risk of shoulder injury in overhead athletes [10,11,12]. 

One of the most common shoulder overuse injuries or disorders in overhead athletes is subacromial pain syndrome. The etiology of subacromial pain syndrome is multifactorial, with narrowing of the subacromial space and/or pathology initiating from the rotator cuff tendons such as the supraspinatus tendon [13]. Tendon pathology may lead to increased blood flow, hypoechoic areas in the tendon, and diffuse hypoechoic thickening of the supraspinatus tendon. The size of the subacromial space can be quantified by various imaging techniques, such as the distance from the acromion to the humeral head, referred to as the acromio-humeral distance (AHD), and some studies have found that AHD is linked to supraspinatus tendon size [7,14]. Interestingly, reduced AHD is associated with various rotator cuff pathologies [15,16] or glenohumeral internal rotation deficit (GIRD) among overhead athletes [17,18] or scapular dyskinesia among adolescent tennis players [19]. In contrast, asymptomatic overhead athletes and wheelchair users show greater supraspinatus tendon thickness and AHD than controls [7,14,20,21], and sometimes also compared with the nondominant side [7,21]. Moreover, shoulder asymptomatic wheelchair users show greater supraspinatus tendon thickness and AHD size than wheelchair users with rotator cuff injury [14]. It has been previously demonstrated that habitual loading and resistance exercise induce tendon hypertrophy of the patellar and Achilles tendon, which reduces tendon mechanical stress for a given load and thereby theoretically lowers the risk of injury [22,23,24,25,26]. However, it is still largely unknown if hypertrophy of the supraspinatus tendon-muscle observed as a greater AHD is a physiological adaptation among badminton athletes, and whether AHD is linked to strength in non-injured shoulders. 

This study aimed to investigate AHD and rotational isometric strength side-to-side in non-injured shoulders of national team badminton players and see if any correlations existed between AHD and strength. The hypothesis was that AHD would be greater on the dominant side compared with the nondominant side as a result of the habitual loading. Secondly, we also hypothesized that a greater AHD on the dominant side was related to a greater ER strength on the dominant side. 

## 2. Materials and Methods

### 2.1. Subjects

Nine adults, 3 female and 6 male national team badminton players, consented and volunteered to participate in the study as a part of the preseason screening in 2006. All participants had been competing at the elite level (highest league) for more than 5 years. Participant consent was given in accordance with the policy statements of American Colleges of Sports Medicine and the Danish Society of Sports Medicine and the Helsinki declaration. The authors had no conflicts of interest. Exclusion criteria included any previous or current injury that could affect the shoulder function. To ensure that the participants were free of shoulder symptoms and pathology, a screening evaluation of the spine and shoulder was performed. The exclusion basis was a positive response in the following test: Hawkins’ test, Jobe’s test, Apprehension test, O´Brien´s test and/or Spurling´s test and foraminal compression/distraction test. Furthermore, a thorough sonographic evaluation of the shoulder was performed. Participants with hypoechoic and Doppler findings were excluded. One of the authors conducted both functional and sonographic evaluation verified by the national team physician. Two participants (1 male, 1 female) were excluded from the study based on hypoechoic findings during the sonographic evaluation. Therefore, the study ended up with 7 participants: 2 females and 5 males. Player characteristics are shown in Table 1.

### 2.2. Acromiohumeral Distance (AHD)

**Figure 1:**
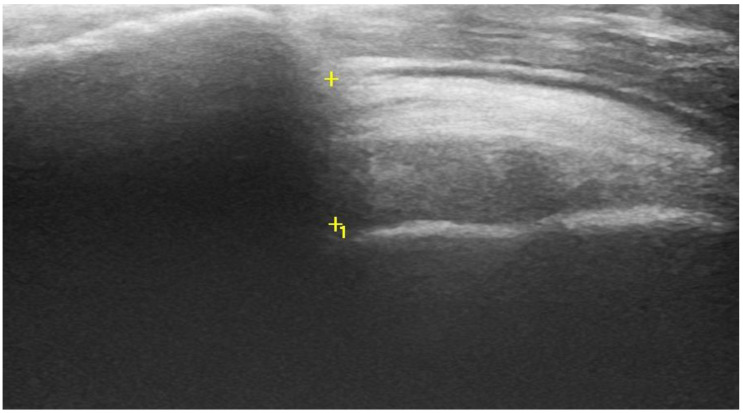
Example of ultrasonographic image of the acromio-humeral distance (AHD) at 0° of abduction. The measurement was the distance between the +/+1 marks.

All participants were instructed to refrain from any rigorous shoulder activity 24 h prior to all examinations including AHD scanning to avoid influence of tissue changes and swelling [27]. A portable GE Medical Systems Logic e dynamic real-time ultrasound (US) scanner with a 13–6 MHz linear transducer 5 cm probe (GE Medical Systems, Milwaukee, WI, USA) was used to obtain the ultrasound (US) images. Preset default parameters were used for musculoskeletal shoulder settings. The AHD scanning was performed with the shoulder in 0 degrees of abduction and humerus in a neutral position. During the ultrasound scanning, the participant’s position was standardized in the following way: Participants were carefully instructed to sit upright without back support with their feet flat on the ground and their arms along their body with the ulnar side of their hand on their thighs and the thumbs pointing upward [21]. In this position, the subjects’ arms were relaxed or supported, and thereby, any activity from humeral head depressors on the AHD was mitigated. The US transducer was placed in the frontal plane parallel to the longitudinal axis of the humerus and positioned at the shortest possible tangible distance between the hyperechoic landmarks of the most superimposed part of the humerus and acromion on the US screen [19,21,28]. The same examiner obtained three US images from each side of the participants. Another researcher, unaware of the side dominance, measured and calculated the AHD based on the obtained images. The AHD was defined as the shortest linear distance from the hyperechoic landmark of the humeral head and below the lateral edge of the acromion, where the subacromial soft tissue emerges from the acoustic shadow of the acromion, see Figure 1. 

### 2.3. Shoulder Rotational Strength

Measurements of shoulder strength were obtained with an HHD (J-Tech Power Track^®^ dynamometer, JTECH Medical, Salt Lake City, UT, USA) as previously described [9]. Shoulder ER and IR strength tests were performed in the supine position with the arm at 90° of abduction and 0° of rotation in the scapular plane [6,9]. The elbow was flexed at 90° and the examiner stabilized the humerus by pressing it down toward the examination table. The subject was allowed to grasp the table with the non-testing arm to provide more stabilization. This position was chosen because of its resemblance to the badminton smash and to obtain maximal strength values with the minimum risk of a shoulder injury [6,9,29]. The testing angle was checked by visual inspection. For the ER-strength test, the player externally rotated the shoulder against the HHD, while the HHD was located proximal to the ulnar styloid processus. For the IR-strength test, the player internally rotated the shoulder against the HHD, while the HHD was located proximal to the ulnar styloid processus. The isometric “make test” consisted of a 5–6 s maximal effort by the subject. The HHD was calibrated prior to each test. One examiner performed all the tests. 

The subject warmed up by performing 15 repetitions of both ER and IR movements, separately, with a 1 kg dumbbell in sideline position before the strength tests without exceeding fatigue. The subject was instructed in a standardized manner not to move other body parts during the testing procedures. A standardized verbal encouragement was given during the effort. Subjects were informed on the importance of exerting a maximal effort prior to the trial. The mean of three maximal voluntary efforts was recorded and calculated for IR and ER, respectively. To reduce the effects of fatigue, a 20–30-s rest [9] was given between the three efforts. The order of testing in the present study remained constant (ER right, IR right, ER left and IR left) [9]. Within session test–retest reproducibility has previously been assessed in our lab [9]. The typical error in percentage for IR and ER strength assessments were 4.7% and 5.8 %, respectively. There were no systematic differences between the two highest values for either IR or ER strength testing. 

### 2.4. Statistical Analysis

The statistical analysis was performed using the statistical analysis software package GraphPad Prism© 7 (San Diego, CA, USA). Paired *t*-test was used to examine side differences (dominant vs. nondominant side) assuming normal distribution. Pearson’s correlation coefficient was used to calculate correlations. The statistical significance was set at *p* < 0.05. Physical characteristics are presented as mean with standard deviation (mean ± SD). Descriptive data are presented as mean with standard error of mean (mean ± SEM). Torque is shown as force (N) multiplied by forearm length (m) = Nm.

## 3. Results

**Figure 2:**
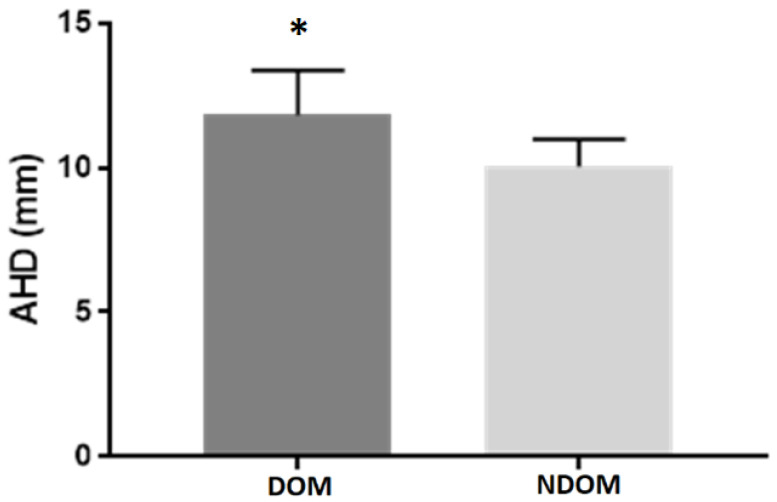
AHD * *p* = 0.018 difference between dominant and nondominant side. Values are mean ± standard error of mean (SEM).

Data on AHD, shoulder strength and strength-ratio are presented in Table 2. AHD was greater on the dominant side compared with the nondominant side (*p* = 0.018) (see Figure 2). IR strength was greater on the dominant side compared with the nondominant side (*p* = 0.041). No other side–side differences were demonstrated.

**Figure 3:**
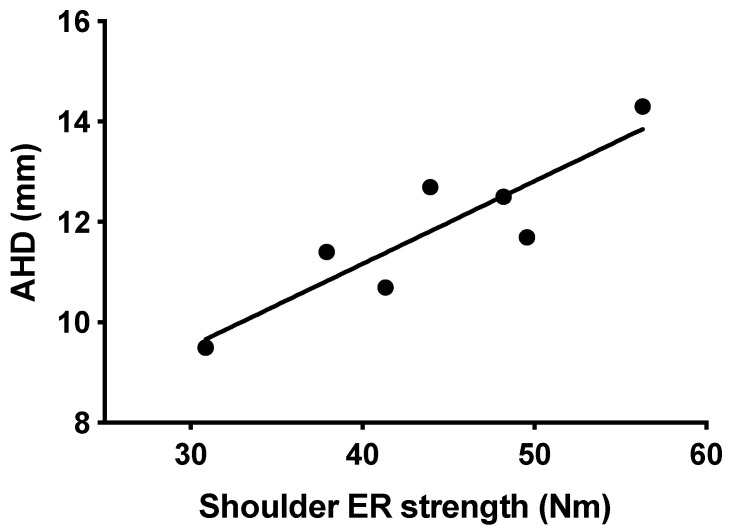
*p* = 0.007 correlation, *r* = 0.900, between AHD and external rotation (ER) strength on the dominant side.

There was a very high correlation between AHD and ER strength (*p* = 0.007, *r* = 0.900) on the dominant side, Figure 3. A correlation was also shown between AHD and the ER/IR strength ratio on the dominant side (*p* = 0.033, *r* = 0.793). 

## 4. Discussion

This study aimed to profile the acromio-humeral distance (AHD) together with shoulder isometric strength in injury-free shoulders of national elite badminton players. The main findings were that AHD was greater on the dominant side compared to the nondominant side. Moreover, IR strength was greater on the dominant side compared to the nondominant. Interestingly, a very strong correlation was demonstrated between AHD and ER strength on the dominant side. Furthermore, a correlation was also shown between AHD and the ER/IR strength ratio on the dominant side. These data are the first to suggest that a greater shoulder ER strength is strongly associated with greater AHD, indicating physiological hypertrophy of the supraspinatus tendon-muscle that reduces the tendon mechanical stress for a given load likely as a sports-specific adaptation that could lower rather than increase the risk of injury. These novel data also suggest that strengthening of the shoulder external rotators could be a potential strategy to reduce the risk of future shoulder injury.

### 4.1. AHD

Several investigators have examined AHD in overhead athletes with conflicting results. A greater AHD on the dominant side compared to the nondominant side has been reported in healthy female overhead athletes (handball, water polo, badminton, squash) [21] and in volleyball players of both genders with and without subacromial pain syndrome (SAPS) [7]. Moreover, a greater AHD has also been demonstrated in asymptomatic volleyball players, baseball players and wheelchair users compared to healthy matched controls [7,14,20]. Recently, Fournier et al. found that both AHD and supraspinatus tendon thickness in manual wheelchair users were greater compared to healthy controls. These authors suggested that the greater AHD/ supraspinatus tendon thickness of manual wheelchair users without shoulder pain is tendon hypertrophy as a sports-specific physiological adaptation to increased load. Indeed, they found that the supraspinatus tendon occupies a larger part of the outlet (occupation ratio; supraspinatus tendon to AHD) among the manual wheelchair users than healthy able-bodied controls [14]. In contrast, an AHD smaller on the dominant side than on the nondominant side has been found in overhead athletes of both genders (handball, water polo, badminton, squash, and volleyball) with glenohumeral internal rotation deficit (GIRD) on the dominant side [17,18]. A lower AHD has also been reported in junior elite tennis players compared to healthy matched controls. However, 43.4% of the tennis players had scapula dyskinesia compared with 20% in the control group [19], suggesting that shoulder dysfunction (GIRD or scapula dyskinesia) negatively impacts AHD size [17,19]. Nevertheless, it is unclear whether tendon pathology, such as hypoechoic findings that give rise to tendon swelling, could have affected the AHD results. This has not been reported in the aforementioned studies [7,17,19,20,21]. Pathological tendon tissue increases the CSA (cross sectional area)/thickness of the tendon [30] and in the present study the adult national badminton players did not show any sign of injury or pathology (hypoechoic area and Doppler activity) when a highly experienced clinician examined their shoulder and rotator cuff clinically and with ultrasound imaging. In the present study, the AHD size was 18% greater on the dominant side than the nondominant side, which could indicate a physiological hypertrophy response of the supraspinatus tendon due to the increased demands on the throwing/racket arm. It has been previously demonstrated that habitual loading and resistance exercise induce tendon hypertrophy in the lower extremity such as the patellar and Achilles tendon, which reduces tendon mechanical stress for a given load and thereby theoretically lowers the risk of injury [22,23,24,25,26,31]. Wang et al. suggest that hypertrophy without hypo echogenicity of the supraspinatus and bicep tendons, together with an increased AHD, should be considered as a normal physiological profile among overhead athletes [20]. Moreover, Ocguder et al. believe that increased thickness of the supraspinatus tendon is a sports-specific adaptation among overhead athletes [32]. So, together, this may also be true as in the present study a greater AHD size very likely reflects supraspinatus hypertrophy reflecting a sports-specific adaptation that lowers supraspinatus tendon mechanical stress and thereby theoretically reduces rather than increases the risk of injury. Moreover, the greater tendon hypertrophy probably occurred by new collagen added to the periphery of the tendon [33] as a rodent study has suggested that tendon growth primarily occurs from the superficial layers [34]. 

### 4.2. Shoulder Rotational Strength Profile

A higher IR strength on the dominant side compared with the nondominant side has been reported in adolescent elite badminton players [9]. A lower eccentric ER strength to concentric IR strength ratio on the dominant side compared with the nondominant side also has been reported in male badminton players [8] and by our lab in an unpublished study by Stausholm suggesting that badminton players, in general, follow the same pattern of muscle imbalance as other overhead sports [5,6,8,9]. In the present study, a higher IR strength but not a lower ER/IR strength ratio were found on the dominant side (0.83) compared to the nondominant side (0.91), likely reflecting the small sample size. It should be noted that a greater IR strength on the dominant side without a corresponding greater ER strength has been documented in many overhead sports and is commonly regarded as a sport-specific adaptation [5,6,7,8,9]. Moreover, in most studies, external rotation weakness is associated with an increased risk of shoulder injury in overhead athletes [10,11,12,35].

### 4.3. Correlation between AHD and ER Strength

Leong et al. have previously demonstrated a positive correlation between AHD and supraspinatus tendon thickness, ER-strength and the ER/IR strength ratio across all participants both in baseball players with and without subacromial pain syndrome (SAPS) and healthy matched controls. However, it should be mentioned that their study did not report if any ultrasound scan had been performed prior to AHD measurements in order to detect any shoulder joint–tendon pathology such as hypoechoic sonographic findings that could have affected the results [7]. Moreover, Leong et al. used the AGT method (acromio-greater tubercle distance) to measure AHD. This method does not measure the tangential distance from the edge of acromion to the humerus, as in the present study. Our study demonstrated a strong positive correlation between AHD and ER-strength, with a correlation coefficient that was much greater than what was found by Leong et al. [7] suggesting that the present data is maybe a representation of the relationship between physiological hypertrophy of the supraspinatus tendon-muscle (AHD size) and ER strength. Moreover, a correlation was also shown between AHD and the ER/IR strength ratio on the DOM side. Collectively, these data suggest that physiologic hypertrophy may also occur for the supraspinatus tendon-muscle (AHD largely represents supraspinatus size) as a result of habitual loading overhead in national elite badminton players.

### 4.4. Limitations

The present study has several limitations. The small sample size could have affected the results. Further, it must be mentioned that several structures may influence the AHD, with the supraspinatus tendon as the greatest structure, and that bursa and fat only contain a smaller part in a healthy condition [36,37]. However, in future studies, this has to be investigated in larger cohorts. Future studies would also need to confirm the present findings with larger sample size correlating strength with AHD size and an isolated supraspinatus tendon thickness measurement, and the risk of tendon injury in a prospective design. Moreover, the present study was designed as a within-subject control model (internal control design) without a control group. However, it has been shown that there are no side–side differences in healthy controls for AHD [38].

### 4.5. Practical Applications

It has been shown that progressive resistance training or habitual loading can induce Achilles and patellar tendon hypertrophy that lowers the mechanical stress tendon for a given load [23,24,25,26,32]. However, the present study is the first to demonstrate that shoulder ER strength is strongly associated with AHD size, largely reflecting supraspinatus tendon-muscle hypertrophy as a result of sport-specific adaptation in national elite badminton players with asymptomatic shoulders. Further, the greater supraspinatus- rotator cuff tendon size may serve to lower the tendon mechanical stress and thereby potentially reduce the risk of injury, which warrants strengthening of the shoulder external rotators as a potential strategy to reduce the risk of future shoulder injury. Several studies have shown that a weak ER or supraspinatus strength are associated with a higher risk of shoulder injury [10,11,12].

## 5. Conclusions

AHD was greater on the dominant side than the nondominant side in injury-free shoulders of national elite badminton players. A strong correlation was also demonstrated between AHD and shoulder external rotational (ER) strength on the dominant side. These first data suggest that physiologic hypertrophy can occur in the supraspinatus tendon, likely as a result of sport-specific adaptation due to habitual overhead loading of the shoulder over a longer time that may serve to lower the tendon mechanical stress and thereby potentially reduce the risk of injury.

## Figures and Tables

**Table 1:** Physical characteristics.

Characteristics	National Badminton Players (*n* = 7)							
	Male (*n* = 5)					Female (*n* = 2)		
	Player 1	Player 2	Player 3	Player 4	Player 5	Player 1	Player 2	Mean ± SD
Age (years)	28	20	31	22	23	21	22	24 ± 4
Weight (kg)	75	87	78	71	85	69	75	77 ± 7
Height (m)	1.82	1.95	1.81	1.85	1.89	1.73	1.83	1.84 ± 0.07
BMI (kg/m^2^)	23	23	23	21	24	23	23	23 ± 1
Forearm length (m)	0.26	0.28	0.26	0.26	0.27	0.24	0.25	0.26 ± 0.01

Values are mean ± SD. Male, female. Age (in years). Weight is shown in kilograms (kg). Height and forearm length is shown in meters (m).

**Table 2:** Descriptive data.

Measurements	National Badminton Players (*n* = 7)							
	Male (*n* = 5)					Female (*n* = 2)		
	Player 1	Player 2	Player 3	Player 4	Player 5	Player 1	Player 2	Mean ± SEM
AHD (mm)								
Dominant	13	12	14	11	13	11	10	11.8 ± 0.6 *
Nondominant	10	11	11	10	9	10	9	10 ± 0.4
ER strength (Nm)								
Dominant	44	50	56	41	48	38	31	44 ± 3
Nondominant	38	52	57	47	49	32	32	44 ± 4
IR strength (Nm)								
Dominant	56	70	50	53	58	43	46	54 ± 3 #
Nondominant	50	57	53	50	55	39	34	48 ± 3
ER/IR strength-ratio								
Dominant	0.78	0.70	1.12	0.78	0.84	0.89	0.67	0.83 ± 0.06
Nondominant	0.77	0.92	1.06	0.94	0.89	0.83	0.97	0.91 ± 0.04

Values are mean ± Standard error of mean (SEM). * *p* = 0.018, # *p* = 0.041; dominant side significantly different from the nondominant side.

## Data Availability

The data that support the findings of this study are available from the corresponding author upon reasonable request.
